# Case Report: Anesthesia for emergent gastroscopy in a pediatric patient with Bartter’s syndrome

**DOI:** 10.3389/fmed.2026.1824155

**Published:** 2026-05-29

**Authors:** Anyi Lu, Kai Qin, Min Zhong, Chaokun Zeng

**Affiliations:** 1 Department of Anaesthesiology, The Second Affiliated Hospital of Guangzhou University of Chinese Medicine (Guangdong Provincial Hospital of Chinese Medicine), Guangzhou, Guangdong, China; 2 The Second Clinical College of Guangzhou University of Chinese Medicine (The Second Affiliated Hospital of Guangzhou University of Chinese Medicine), Guangzhou, Guangdong, China

**Keywords:** anesthesia, Bartter’s syndrome, case report, emergent, pediatric

## Abstract

Bartter’s syndrome (BS) is a rare inherited disorder characterized by salt-wasting alkalosis and hypokalemia. Perioperative management of BS patients remains limited, with most of them undergoing elective surgery. We presented a case report about a 12-year-old BS child who underwent an emergent gastroscopy due to acute gastrointestinal bleeding. The patient was presented to the operation room with hypokalemia, hyponatremia, severe anaemia, and hemorrhagic shock. The gastroscopy was performed under general anesthesia, and the patient was hemodynamically stable throughout the whole process. Finally, the patient was discharged with no complications. The perioperative challenges of BS patients mainly come from the dramatic metabolic disturbances. The perception of the pathophysiology of BS and the patient-based status may help to recognize risks and thus minimize the morbidity and improve outcome, even in an emergent situation.

## Introduction

Bartter’s syndrome is a rare congenital disease, and the prevalence is about 1/1000000 (1). It is mainly caused by impairment of the thick ascending limb of Henle’s loop, which is responsible for the absorption of sodium, chloride, and potassium (2). The clinical presentations include polyuria and hypovolemia. Children with BS often get poor growth and pubertal delay. Laboratory tests mostly indicate electrolyte disturbance with hypochloremia, metabolic alkalosis, and hypokalemia. The diagnosis can be confirmed by the detection of pathogenic genes responsible for BS in the genetic test. Treatment of BS includes supplementation of fluid, NaCl, and potassium (3). As hypokalemia is common in BS and will lead to complications, for example, muscle weakness and arrhythmia, potassium supplements are required to maintain the target potassium level, mostly ≥ 3 mmol/L. Supplementation of other electrolytes, such as sodium chloride, magnesium, and calcium, depends on clinical and laboratory tests (4). Non-steroidal anti-inflammatory drugs (NSAIDs) treatment is suggested for symptomatic patients who have an inadequate response to NaCl and fluid supplementation. Indomethacin, ibuprofen, and celecoxib are commonly used. NSAIDs are effective in decreasing prostaglandin levels and further reducing urinary secretion of NaCl and potassium (5).

Published experience with anesthesia in BS is confined to case reports, with most of them undergoing elective surgery. In adult elective cases, preoperative potassium was partially corrected and anesthesia proceeded without major complications under general or regional techniques (6–9). Notably, Higa et al. (6) reported a safe general anesthesia in an adult BS patient undergoing elective rectal resection, with potassium level of 1.6 mmol/L just before the induction. Among pediatric cases, Kannan et al. (10) successfully managed an 8-year-old boy for elective orchidopexy under caudal epidural analgesia following preoperative electrolyte correction. Bhaskar et al. (11) reported uneventful sevoflurane anesthesia in an 8-year-old girl for laparoscopic cholecystectomy, and Raza et al. (12) described a 19-month-old infant undergoing elective hiatal hernia repair under ketamine-based induction. Across all prior reports, procedures were elective and allowed preoperative electrolyte optimization. The present case is, to our knowledge, the first to describe emergent anesthesia in a pediatric BS patient with active upper gastrointestinal bleeding and pre-induction potassium of 1.79 mmol/L representing a unique and previously unreported clinical scenario.

## Case report

A 12-year-old male child, with body weight of 28 kg, height of 132 cm, was admitted to our hospital with chief complaint of persistent hematemesis and melena for 1 month and fatigue and pale for 2 days. 12 years ago, he was diagnosed with BS type III (CLCNKB gene mutations) in his 3-month-old due to hypokalemia. Although daily administration of indomethacin, spironolactone, and potassium supplement, his hypokalemia presented recurrently. 1 month ago, he was sent to another hospital due to a cough and hematemesis, and the laboratory test indicated moderate anaemia with hemoglobin of 69 g/L, severe hypokalemia with potassium level of 1.43 mmol/L, and hyponatremia with sodium level of 127.1 mmol/L. Budd-Chiari syndrome or cirrhosis was suspected based on abnormal hepatic transaminases and fibrosis index, portal hypertension by splenomegaly, and intrahepatic biliary dilatation through imaging. Treatments such as defibrotide and portosystemic shunt were suggested. The parents opted for conservative management, and the patient’s clinical status subsequently remained stable.

Two days before admission, the patient appeared pale and fatigued and passed one episode of coffee-colored stool, after which he was admitted to the hospital. On admission, the patient appeared pale and tired. His vital signs were present as body temperature of 36.9 C°, pulse of 125 beats per minute, respiratory rate of 24 per minute, and blood pressure of 94/61 mmHg. The initial laboratory test was worse than before, with hemoglobin of 44 g/L, hypokalemia with potassium level of 1.99 mmol/L, hyponatremia with sodium level of 120 mmol/L, and hypochloraemia with chloride level of 85.9 mmol/L. The fecal occult blood test was positive with 4 + . After 6 h, he suddenly vomited with approximately 50 ml of dark red blood containing visible blood clots. He was conscious but appeared agitated, with low blood pressure of 87/57 mmHg. Considered of acute gastrointestinal bleeding, he was sent to the operation room and underwent emergent endoscopic hemostasis under general anesthesia.

At the arrival of the operating room, pulse oximetry, ECG, and non-invasive blood pressure were measured. ECG demonstrated sinus tachycardia at 137 beats per minute without arrhythmia. His blood pressure was 96/50 mmHg. After 3 min preoxygenation with 100% oxygen using a face mask, anesthesia induction was performed with intravenous injection of propofol 80 mg, sufentanil 8 ug, and cisatracurium 5 mg. Endotracheal intubation was achieved using an endotracheal tube with an internal diameter of 6.0 mm. The mechanical ventilation was performed with the maintenance of the end-tidal CO2 pressure between 35 and 45 mmHg by adjusting tidal volume (6–8 mL/kg) and respiratory rate (15–20/min). Anesthesia was maintained in total intravenous drugs with propofol and remifentanil, which dosage was guided by the Narcotrend index and heart rate. The central venous catheterization and radial arterial catheterization were then established, and the invasive blood pressure and central venous pressure (CVP) were monitored. Continuous 5-lead ECG monitoring was maintained throughout the procedure with vigilance for any emergence of malignant arrhythmia. Arterial blood gas analysis (ABG) was performed to provide real-time electrolytes levels to guide supplementation. 5% glucose and sodium chloride solution (GNS), Ringer’s lactate, 4 U red blood cells, and potassium supplements infused at 21.6 mg/kg/h were given as intravenous infusion through three venous accesses.

He was placed in the left lateral position for gastroscopy. Variceal veins were observed in the esophagus and stomach, with a single ruptured vein near the cardia at the fornix, but without active bleeding. Endoscopic variceal ligation and sclerotherapy were performed, and the hemodynamics remained stable without arrhythmia occurred during the procedure. The duration of the procedure was 27 min. The ABG showed potassium level increased from 1.79 to 2.01 mmol/L, sodium level increased from 121.1 to 122.6 mmol/L, and chloride level increased from 94.9 to 96.4 mmol/L. The total urine output was 500 ml. The patient was transferred to the ICU with endotracheal intubation, and he was extubated and transferred to the pediatrics wards after 2 days. Despite the continuous intravenous and oral potassium supplementation after surgery, serum potassium remained persistently low, fluctuating between 1.55 and 2.00 mmol/L. The patient was discharged without complication 8 days later, with a potassium level 1.87 mmol/L. The timeline of important clinical events was present in Table 1 and the CARE checklist was provided in Supplementary material.

**TABLE 1:** Timeline of clinical events.

Time	Event
Infant	Diagnosed with Bartter’s syndrome due to hypokalemia.
1 month ago	Cough and hematemesis, suspected Budd-Chiari syndrome or cirrhosis, stable after conservative treatment.
Emergency admission	Pale and hematemesis for 2 days
6 h after admission	Vomited with approximately 50 ml of dark red blood containing visible blood clots.
Preoperative laboratory results	Hemoglobin 44 g/L, potassium level 1.99 mmol/L, sodium level 120 mmol/L, chloride level 85.9 mmol/L.
Emergency intervention	Gastroscopy performed under general anesthesia
Follow-up	No complications and discharged after 1 week

## Discussion

Bartter’s syndrome (BS) is a rare congenital renal tubular disease characterized by metabolic alkalosis, hypokalemia, and hypochloraemia. For BS patients, the compromised fluid and electrolyte homeostasis may further be destabilized by the physiological stress of surgery and anesthesia, which poses a significant challenge for anesthesiologists. This is particularly true in the present case, where active UGIB, hemodynamic instability, and the emergent nature of the procedure precluded standard preoperative electrolyte optimization, demanding risk-benefit decision-making under severe hypokalemia.

Gastrointestinal bleeding in children is uncommon but potentially critical, especially upper gastrointestinal bleeding (UGIB). UGIB is defined as bleeding originating from proximal to the ligament of Treitz, including the esophagus, stomach, and duodenum. UGIB often presents with hematemesis, less with melena and emesis. The mortality for pediatric UGIB ranges from 5% to 15% in worldwide, with different etiology (13). In our case, the patient was diagnosed as BS type III in his young infant period due to hypokalemia, and he received daily spironolactone, indomethacin, and potassium supplementation. Also, his imaging exam indicated suspected Budd-Chiari syndrome or cirrhosis. Both variceal and non-variceal causes may contribute to his brisk bleeding and critical hemorrhagic shock course. Delaying the procedure to allow electrolyte optimization was not a viable option in this case, as the patient presented with active UGIB and hemodynamic instability, and the risks of further hemorrhage, progressive hemodynamic deterioration, and aspiration from repeated hematemesis were judged to outweigh the arrhythmic risk of proceeding under severe hypokalemia. Also, the patient presented the persistent and severe hypokalemia after daily treatment, showing that this chronic condition may be difficult to correct completely in a short time. Vitez et al. (14) demonstrated no increase in intraoperative dysrhythmias in patients with chronic hypokalemia. Higa et al. (6) also reported a safe and successful general anesthesia without arrhythmic consequence in an adult patient with a lower potassium of 1.2–2.1 mmol/L. In the present case, the patient underwent urgent endoscopy with the potassium level of 1.79 mmol/L, and the potassium level remained low, with the highest level of 2.01 mmol/L, even with continuous supplementation of potassium under serial arterial blood gas guidance. No intraoperative arrhythmia occurred under the continuous 5-lead ECG monitoring throughout the procedure. The perioperative safety of potassium levels lower than 2.0 mmol/L for chronic hypokalemia remains unknown and requires further evidence support, as the potassium level ranged from 2.1 to 3.4 mmol/L in most reported cases. Nevertheless, the present case suggests that in emergent situations where preoperative correction is not feasible, proceeding under severe chronic hypokalemia may be acceptable provided that continuous ECG surveillance, invasive hemodynamic monitoring, and intravenous potassium replacement are employed as mitigating strategies.

Anesthetic techniques depended on the type of surgery and the patient-based risk assessment. Endoscopy for evaluation and treatment is recommended within 24–48 h of acute and severe bleeding (15), and in our case, emergent endoscopic hemostasis was therefore performed given the active UGIB and hemodynamic instability. The complication occurred in about 2.5%, and hypoxia was the most common complication in pediatric endoscopy. General anesthesia is recommended in endoscopy for children with GI bleeding (16, 17). In our case, general anesthesia with tracheal intubation was chosen rather than deep sedation for several reasons. First, the patient presented with active hematemesis and hemodynamic instability, creating a high risk of pulmonary aspiration, which tracheal intubation provided definitive airway protection that deep sedation or supraglottic airway devices could not guarantee. Second, hypokalemia induces weakness of smooth muscle, delayed gastric emptying, and consequently increases the risk of aspiration. Hypokalemia may exacerbate after anesthesia induction due to the stress response and activation of beta-adrenergic receptors. Third, the hemodynamic instability precluded the use of standard sedation doses, as respiratory depression without airway control would have been particularly hazardous.

For the induction and maintenance, propofol was chosen for its titratable, short-acting profile, anti-emetic properties which may be beneficial in a high aspiration-risk patient. Sufentanil was selected over other opioids for its favorable hemodynamic stability at reduced doses and predictable pharmacokinetics. This patient weighed only 28 kg at 12 years of age, reflecting the chronic growth retardation characteristic of long-standing BS type III. Drug dosing was therefore based on actual body weight rather than age-expected weight. The induction dose was deliberately reduced to minimize cardiovascular depression in the context of severe anaemia, hemorrhagic shock and reduced physiological reserve in this malnourished child. There are highly variable anesthetic practices of BS patients, with evidence confined to a few case reports (18). The controversy in volatile anesthetics remained due to the concern of cardiovascular suppression and potential hypotension (10), while some cases managed with isoflurane free of complications (6, 10, 19). Cisatracurium was chosen because its Hofmann elimination renders it entirely independent of renal and hepatic metabolism, which was critical in a patient with renal tubular dysfunction (BS type III) and suspected hepatic disease. Furthermore, hypokalemia potentiates non-depolarizing neuromuscular blockade by reducing the resting membrane potential of muscle fibers, increasing the risk of prolonged neuromuscular blockade and residual muscle weakness. Tracheal intubation with mechanical ventilation therefore provided essential airway protection and guaranteed adequate ventilation throughout the procedure, safeguarding against the risk of respiratory compromise from potentiated neuromuscular blockade in this severely hypokalaemic patient. The patient remained stable through the procedure, without complications of hypoxia and hypotension.

For electrolytes management of BS patients, the guideline for perioperative management of people with inherited salt-wasting alkaloses recommends that preoperative assessment should include the type of surgery, the time course of BS, concurrent medications, electrolyte disturbance, and risk factors. It is suggested that serum potassium of 2.8–3.0 mmol/L with magnesium ≥ 0.5 mmol/L was acceptable in stable patients with minor procedures. A higher acceptable level of potassium (3.3 mmol/L) was recommended for those with hypomagnesemia (< 0.5 mmol/L), as it would increase the dysrhythmic risk of hypokalemia. The non-urgent surgery should be rescheduled if the patient fails to meet the acceptable level of electrolytes, and referral to an expert physician may be beneficial for professional management advice (18). In our case, potassium supplementation was administered via central venous catheter at 21.6 mg/kg/h. This rate was selected to achieve a meaningful increment in serum potassium within the duration of the procedure while remaining within accepted safe infusion limits via central venous access. Serial arterial blood gas analysis was performed to guide real-time dose titration, with serum potassium rising from 1.79 to 2.01 mmol/L by the end of the procedure. Serum magnesium levels were not measured intraoperatively, which we acknowledge as a limitation, given that hypomagnesemia impairs renal potassium conservation and increases the risk of refractory hypokalemia and arrhythmia. Although no symptomatic arrhythmia occurred, intraoperative magnesium monitoring should be considered in future cases of BS undergoing emergent anesthesia.

Despite the continuous potassium supplementation after surgery, serum potassium remained low than 2.0 mmol/L. This reflects the chronic and refractory nature of renal tubular potassium wasting inherent to BS type III, in which normalization of serum potassium is rarely achievable despite aggressive supplementation. Importantly, the patient’s potassium level remained stable throughout the postoperative period without further decline. Given his longstanding tolerance of chronic hypokalemia and clinically stable condition, discharge with a potassium level 1.87 mmol/L was deemed appropriate with optimized oral supplementation and outpatient follow-up.

Several strengths and limitations of this case report should be acknowledged. The strengths include the use of invasive hemodynamic monitoring with arterial and central venous catheters, Narcotrend-guided anesthesia depth titration, and serial arterial blood gas-guided potassium supplementation, all of which contributed to the safe conduct of anesthesia under severe hypokalemia. However, this report has several limitations. First, the choice of intravenous fluids could have been further optimized. Plasmalyte, with a sodium concentration closer to plasma (140 mmol/L) and magnesium content, would have been preferable over Ringer’s lactate in the context of hyponatremia and hypokalemia. Second, serum magnesium levels were not monitored intraoperatively, which is relevant given its role in arrhythmia risk and potassium homeostasis. Finally, a definitive diagnosis distinguishing Budd-Chiari syndrome from cirrhosis was not established due to the emergent clinical context.

In summary, this is the first emergent case of a pediatric BS patient undergoing gastroscopy for UGIB, with general anesthesia. The perioperative challenges of BS patients mainly come from the dramatic metabolic disturbances inherent to the syndrome, which are further compounded in emergent settings where standard preoperative optimization is not feasible. The understanding of the pathophysiology of BS and the patient-based risk-benefit assessment may help to guide clinical decision-making, recognize potential complication and thus minimize the morbidity and improve outcome, even in an emergent situation.

## Data Availability

The original contributions presented in this study are included in this article/Supplementary material, further inquiries can be directed to the corresponding author.
